# Using eHealth to engage and retain priority populations in the HIV treatment and care cascade in the Asia-Pacific region: a systematic review of literature

**DOI:** 10.1186/s12879-018-2972-5

**Published:** 2018-02-17

**Authors:** Julianita Purnomo, Katherine Coote, Limin Mao, Ling Fan, Julian Gold, Raghib Ahmad, Lei Zhang

**Affiliations:** 1 The Albion Centre, South Eastern Sydney Local Health District, 150-154 Albion Street, Sydney, NSW 2010 Australia; 2 Centre for Social Research in Health, UNSW, Sydney, NSW 2052 Australia; 3 Centre for Neuroscience and Trauma, Blizard Institute, Queen Mary University of London, London, UK; 4 Research Center for Public Health, Tsinghua University, Beijing, 100084 China; 5 Melbourne Sexual Health Centre, Alfred Health, Melbourne, Australia; 6 Central Clinical School, Faculty of Medicine, Monash University, Melbourne, Australia; 7 School of Public Health and Preventive Medicine, Faculty of Medicine, Monash University, Melbourne, Australia

**Keywords:** HIV, Treatment cascade, Testing, Key population, Asia-Pacific, mHealth, Mobile phone, Social media, Review

## Abstract

**Background:**

The exponential growth in the reach and development of new technologies over the past decade means that mobile technologies and social media play an increasingly important role in service delivery models to maximise HIV testing and access to treatment and care. This systematic review examines the impact of electronic and mobile technologies in medical care (*eHealth*) in the linkage to and retention of priority populations in the HIV treatment and care cascade, focussing on the Asia-Pacific region.

**Methods:**

The review was informed by the Preferred Reporting Items for Systematic Reviews and Meta-Analyses (PRISMA) statement from the Cochrane Collaboration guidelines. Both grey and published scientific literature from five different databases were searched for all original articles in English published from 2010 to 2017. Studies conducted outside the Asia-Pacific region or not including HIV priority populations were excluded. The methodological quality of studies included in the review was assessed using the Quality Assessment Tool for Quantitative Studies.

**Results:**

The database search identified 7309 records. Of the 224 peer-reviewed articles identified for full text review, 16 studies from seven countries met inclusion criteria. Six cross sectional studies found evidence to support the use of eHealth, via text messages, instant messaging, social media and health promotion websites, to increase rates of HIV testing and re-testing among men who have sex with men (MSM). Evidence regarding the efficacy of eHealth interventions to improve antiretroviral treatment (ART) adherence was mixed, where one randomised controlled trial (RCT) showed significant benefit of weekly phone call reminders on improving ART adherence. Three further RCTs found that biofeedback eHealth interventions that provided estimated ART plasma concentration levels, showed promising results for ART adherence.

**Conclusions:**

This review found encouraging evidence about how eHealth can be used across the HIV treatment and care cascade in the Asia-Pacific region, including increasing HIV testing and re-testing in priority populations as well as ART adherence. eHealth interventions have an important role to play in the movement towards the end of AIDS, particularly to target harder-to-reach HIV priority populations, such as MSM.

**Electronic supplementary material:**

The online version of this article (10.1186/s12879-018-2972-5) contains supplementary material, which is available to authorized users.

## Background

The term *HIV treatment and care cascade* describes the process of HIV testing, linkage to and retention in care, initiation and adherence to antiretroviral treatment (ART), and ultimately, viral load suppression. Achieving the ultimate outcome of the cascade results directly in improved health outcomes for people living with HIV (PLHIV) and reduced risk of HIV transmission [1]. To maximise the benefits of ART, individuals must progress along the continuum of care promptly after HIV infection. HIV testing is a critical first step in linking PLHIV to the HIV treatment and care cascade, where it also provides an important opportunity to reinforce prevention against HIV transmission. Although HIV testing and treatment programme coverage is improving globally since 2011 in the era of *Treatment as Prevention*, disengagement from HIV clinical care at each stage of the treatment and care cascade still occurs. Linkage to and retention in HIV clinical care are critical to achieving universal access to HIV treatment and care with the aim of sustained HIV viral suppression [2, 3].

The roles of electronic health (eHealth) and mobile health (mHealth) in health care are expanding. The World Health Organization (WHO) Global Observatory for eHealth (GOe) defines eHealth as “the use of information and communication technologies (ICT) for health”. This encompasses the delivery of health information for health professionals and health consumers, through the internet and telecommunications [4]. The GOe defines mHealth as “medical and public health practice supported by mobile devices, such as mobile phones, patient monitoring devices, personal digital assistants (PDAs), and other wireless devices” [5]. This paper will use the general term *eHealth* to denote eHealth, mHealth, and ICT, and will refer to specific technologies where applicable.

Accessibility to the internet and mobile phones internationally and particularly in developing economies in the Asia-Pacific is surging [6]. Six in ten people from Asia-Pacific countries subscribed to mobile services, with 1.7 billion smartphone connections at the end of 2015. By 2020, 74% of the region’s population is expected to have a mobile subscription and will add a further 1.3 billion smartphone connections, reaching two-thirds of the region’s total connections [7]. The impact of eHealth on the outcomes of health promotion and health care access and delivery are increasingly examined across a wide range of diseases and health behaviours [5, 8–11]. The benefits of text messaging for improving clinical outcomes and attendance in chronic disease self-management have been well documented [8, 11].

For PLHIV, eHealth is increasingly being integrated into HIV-related disease self-management and service delivery. While short message service (SMS) interventions are evidenced to be effective in enhancing ART adherence [11–14], eHealth overall is considered as the most promising approach to deliver cost-effective interventions along the HIV treatment and care cascade [15–19].

HIV priority populations, groups at increased risk of HIV due to specific higher-risk behaviours [20], are typically harder-to-reach due to stigma and discrimination in relation to HIV and associated behavioural patterns. Research from Asia-Pacific countries has shown that eHealth interventions are considered acceptable and feasible modalities to engage populations ranging from men who have sex with men (MSM), transgender women to female sex workers [21–34].

Based on the aforementioned advantages, global health and development agencies (e.g. WHO and the United Nations Population Fund [UNFPA]) have identified the potential for eHealth in improving HIV outcomes [20, 35]. Existing reviews of eHealth interventions targeting PLHIV focus on the application of specific devices (such as mobile phones [36, 37], telephone [38, 39], and computer [40]) and particular technology functions (such as text messaging [12, 41], internet [42], mobile phone applications (apps) [43], social media [44] and social marketing [45]), across a range of HIV priority populations. However, limited reviews have investigated the use of eHealth in the context of the HIV treatment and care cascade to improve service engagement among HIV priority populations. The most commonly researched HIV priority populations were MSM, with a focus on HIV prevention and testing [42, 45, 46]. Catalani et al. systematically reviewed the published literature and reported a lack of evidence concerning the use of eHealth to address the needs of a range of priority populations, including MSM up to 2011 [47].

The Asia-Pacific covers the World Health Organization (WHO) divisions of the Western Pacific Region and South East Asia Region. These geographically linked regions encompass diversity in cultures and economies, as well significant uptake of mobile technology innovations, particularly in South East Asia [5]. This review focusses on the Asia-Pacific region to inform future HIV policy and programming in these parts of the world.

To our knowledge, no review has yet examined the use eHealth targeting all HIV priority populations across the entire HIV care continuum in the Asia-Pacific region. This systematic review aims to describe and critically examine, the uptake of eHealth interventions applied to engage and retain priority populations in HIV treatment and care, in the Asia-Pacific region.

## Methods

This review followed the PRISMA (Preferred Reporting Items for Systematic Reviews and Meta- Analyses) guidelines [48] and used a guided approach to retrieve relevant studies. The review included all experimental (randomised controlled trials or case-control studies) and observational (cross-sectional or longitudinal) studies that examine the impacts of different types of eHealth interventions in engaging and retaining priority populations in the HIV testing, treatment and care cascade within the Asia-Pacific region. Qualitative studies were excluded from this review.

### Search strategies and selection criteria

The Population, Intervention, Comparison, Outcome (PICO) model was used to develop inclusion criteria and search terms. In consultation with a librarian (LF), a search strategy was developed to locate all relevant studies. Priority populations were the selected *population*, referring to MSM, sex workers (SW), people who inject drugs (PWID) and transgender (TG) people. *Interventions* included: internet (internet-based, web-based and online intervention), social media (e.g. education initiatives, direct messaging, social marketing, partner referrals, linkage to testing sites, delivery of postal tests, etc.), cell phone, text messaging, mobile apps, reminder systems, computer (e.g. personal computer, laptop, handheld and ultra-portable computer, etc.), personal digital assistant (PDA), portable media player, handheld video-games console (e.g. Playstation Portable), telemedicine/telehealth conducted in Asia-Pacific countries. All types of *comparison* and no comparison were incorporated in this review. *Outcomes* comprised of behavioural outcomes (HIV testing uptake, HIV re-testing rates); biomedical outcomes (viral load, CD4+ T-cell counts, HIV-related mortality and morbidity rates); and treatment adherence measurement (pill counts, electronic monitoring, patient self-report, medication diaries, clinic or pharmacy reports).

Combining terms related to HIV, eHealth, HIV treatment and care cascade and priority populations (Table 1), electronic searches for peer-review articles were performed in PubMed, MEDLINE, EMBASE, Cumulative Index of Nursing and Allied Health (CINAHL) and Web of Science. Grey literature was obtained from the: Cochrane Library; World Health Organization Library Information System (WHOLIS); World Bank e-Library; British HIV/AIDS Association (BHIVA); International AIDS Society; International AIDS Conference; and Australasian HIV/AIDS conference databases. The search included a wide range of Medical Subject Heading (MeSH) terms, subject headings, free-text terms with all potential synonyms, related terms and variant spellings using truncation and combinations, keywords and controlled vocabulary search terms derived from review questions. Subject terms were identified through preliminary exploration searches of the above electronic databases.

**Table 1:** List of terms for literature search

Source		Variations of the following search terms were used
PubMed, MEDLINE, EMBASE, CINAHL, Web of Science, Cochrane Library, WHOLIS, World Bank e-Library, BHIVA, International AIDS Society, International AIDS Conference, The Australasian HIV/AIDS conference	HIV infection	HIV, AIDS
	Technology intervention	mHealth, mobile health, cellular phone, cell phone, mobile phone, handphone, smartphone, personal digital assistant, portable media player, handheld video-game consoles, computer, personal computer, handheld and ultra-portable computer, desktop, laptop, palm pilot, netbook, mobile application, SMS, MMS, text messaging, reminder systems, email, instant messaging, chat room, live chat, multimedia, blogging, podcast, social media, Facebook, Twitter, MySpace, YouTube, social networking, internet forums, wireless technology, wi-fi, world wide web, website, internet, online, eHealth, telehealth, telemedicine
	HIV treatment and care cascade	Testing, screen, diagnose, retention, linkage, care, cascade, follow up, counselling, treatment, suppression, PrEP, PEP, ART, medication adherence
	Priority population	MSM, sex workers, people who inject drugs, transgender

Initially the review was limited to published and unpublished studies including *in press* and *in progress* articles between January 2010 and December 2014. A subsequent search was conducted to extend the review period up until June 2017. Studies were excluded if they were not in English, were conducted outside the Asia-Pacific region, or did not include priority populations. In this review article, the term “Asia-Pacific” refers to the following countries: Australia, Bangladesh, Brunei, Cambodia, China, Cook Islands, Fiji, Hong Kong, India, Indonesia, Japan, Kiribati, Korea, Lao People’s Democratic Republic, Malaysia, Maldives, Mongolia, Myanmar, Nepal, New Zealand, Pakistan, Papua New Guinea, Philippines, Sri Lanka, Taiwan, Thailand, Timor-Leste, Samoa, Singapore, Solomon Islands, Tonga, Vanuatu, and Vietnam.

Duplicate publications and publications derived from the same data sources were excluded using a combined strategy of auto and hand-searching methods. Systematic reviews previously performed on these topics were also excluded. The reference lists of key articles were hand-searched to locate additional studies.

Search results from the twelve databases were downloaded into EndNote X7 software. Two EndNote libraries were created, one for testing and linkage into the HIV treatment and care cascade literature and one for retention in the HIV treatment and care cascade literature. Examples of each search strategy conducted in PubMed are presented in Appendix 1 (see Additional file 1). Search terms were modified in other electronic databases.

### Screening and data extraction

After the removal of duplicates, titles and abstracts of identified articles were screened for relevance to this systematic review by two reviewers (JP, KC). When it was not clear whether the abstract met the inclusion criteria, the full text of potentially relevant studies was reviewed. Failure to meet any of the eligibility criteria resulted in exclusion from the review. The number of excluded studies and reasons for exclusion were recorded at each selection stage. In the case of any disagreement, the articles were discussed by the two reviewers (JP, KC), and further dispute was resolved in consultation with an additional two reviewers (LZ, LM).

Data were extracted using a standardised abstraction form by one reviewer (JP) and independently verified by a second (KC). The following information was gathered from each included study: country, study design, study population, sample size, technology mode, the purpose of the study, study description, study duration, key outcomes of interest and results or authors’ conclusions.

### Quality assessment

The methodological quality of all included studies was assessed using the Quality Assessment Tool for Quantitative Studies [49, 50]. This tool enables a global rating of ‘weak’, ‘moderate’ or ‘strong’ measured by potential selection bias, study design, confounders, blinding, data collection methodology and reporting of withdrawals and dropouts. Component ratings were scored according to the criteria specified in the dictionary that accompanies the assessment tool [49].

A meta-analysis was not performed due to the heterogeneity of intervention types, study designs, study populations and outcome variables among the included studies. Instead, a descriptive summary of the findings across studies was created, based on key themes and outcomes.

## Results

**Fig. 1:**
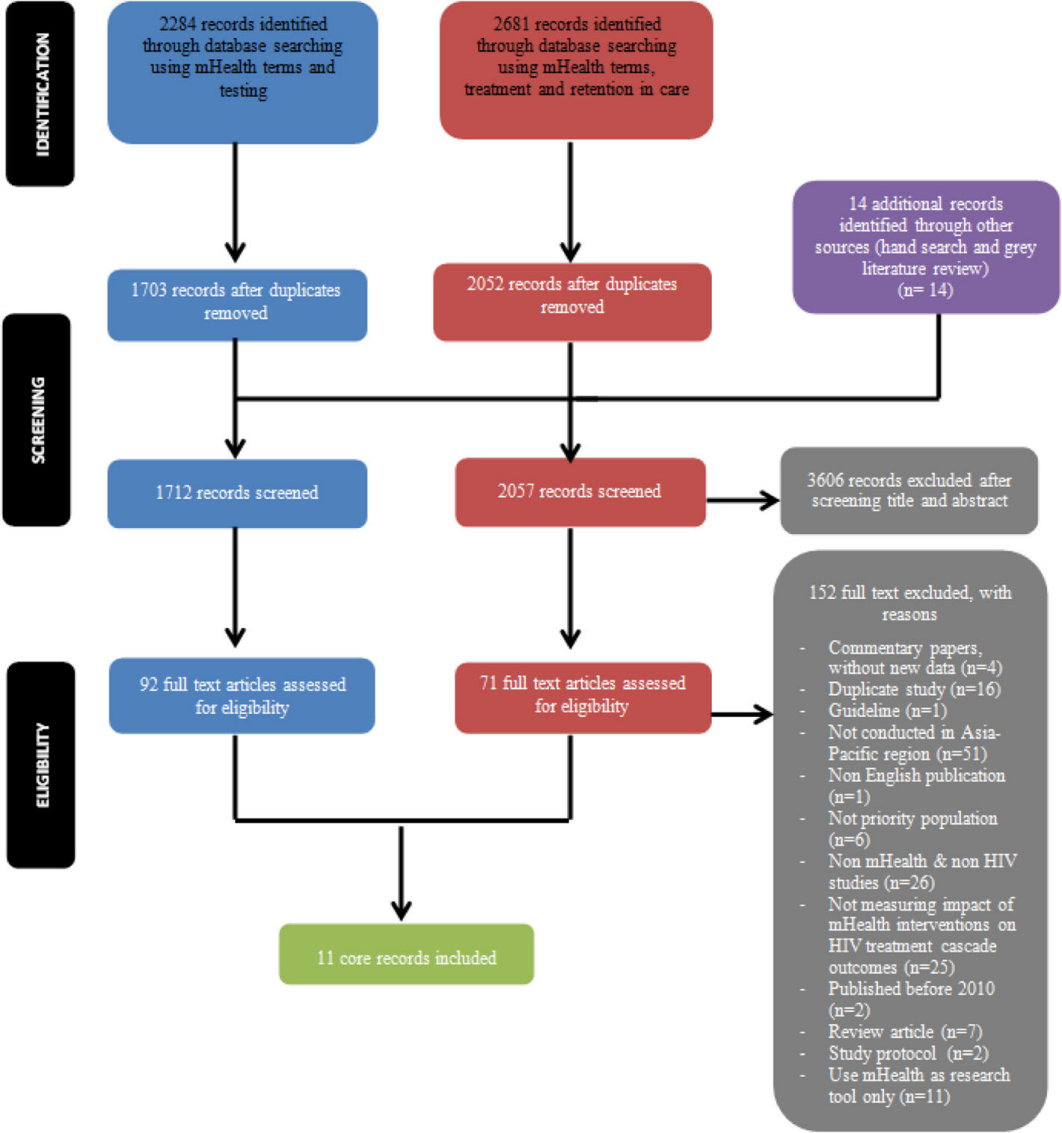
PRISMA Flow Diagram

The initial database search (Fig. 1) identified 4965 articles. On removal of duplicates, 3755 peer review articles remained. Fourteen additional articles from hand-searching and grey literature search were included in the title and abstract review phase. After preliminary screening of titles and abstracts by the reviewers, 163 potentially relevant articles were examined in full against the inclusion and exclusion criteria. Of these, twelve studies [17, 51–61] fulfilled the inclusion criteria. A further article [61] was excluded as it became apparent that it reported on duplicate data from the same data source [59]. The subsequent literature search (to June 2017) found five additional articles [62–66] which met the inclusion criteria, so that the final sample was 16 eligible papers (Tables 2, 3 and 4).

**Table 2:** eHealth and HIV testing and linkage to care

Study	Country	Study design	Study Population Sample size (*N*=)	Technology mode	Purpose of study	Study description	Key outcomes of interest in this review	Results/Authors’ conclusions
*SMS reminders to increase HIV testing and re-testing*								
Bourne et al. 2011	Australia	Cross-sectional	MSM *I* = 714 C = 1084 PI = 1753	SMS	Evaluate impact of SMS reminder system on HIV/STI re-testing rates	SMS testing reminders were sent 3–6 monthly for MSM considered high-risk based on self-reported sexual behaviour. Comparison of HIV/STI re-testing rates among 3 groups.	HIV re-testing rates	Significant increase HIV re-testing rates within 9 months, I (64%) vs. C (30%) (*p* < 0.001) and I (64%) vs. PI (31%) (*p* < 0.001). SMS group was 4.4 times more likely to re-test than Control (95% CI 3.5 to 5.5, *p* < 0.001); SMS group was 3.1 times more likely to re-test than PI group (95% CI 2.5 to 3.8, *p* < 0.001).
*Instant messaging to promote HIV testing*								
Zou et al. 2013	China	Cross-sectional	MSM *N* = 429	Instant messaging	Explore the feasibility of using internet outreach to encourage MSM to get tested for HIV	Two trained MSM volunteers promoted VCT using *active* (instant messaging, chat rooms, mobile phone, email) and *passive* (website banner ads) methods. Those who came for testing completed a survey and HIV/syphilis tests.	HIV testing uptake Motivation for seeking HIV testing Response to internet outreach	Instant messaging was the most effective mode for HIV testing promotion (1:4 men). The email was the least effective (1:140 men). Active internet outreach recruited younger MSM (X^2^ = 11.400, *p* = 0.001), never tested for HIV (X^2^ = 4.281, *p* = 0.039), tested less often (X^2^ = 5.638, *p* = 0.018). Note: Internet effective in encouraging testing but a confounding factor of financial reward.
*Social media campaigns to increase HIV testing in MSM*								
Cheng et al. 2016	China	Cross-sectional	MSM Primary outcome *N* = 22,282 Secondary outcomes *N* = 999	Internet Social media	Evaluate impact of integrated service including internet based prevention services, online-to-offline line service linkage and offline one-stop shop service	Three project components: (1) internet-based prevention services to facilitate HIV prevention; (2) online-to-offline service linkage-online appointments for MSM for HIV testing; and (3) offline one-stop shop service-HIV testing and linkage to care for PLHIV	*Primary outcome:* HIV testing uptake *Secondary outcomes:* linkage to and retention in care	Six years of project implementation, the project accounted for 80% of total HIV tests (22,282/26,884) and new HIV diagnoses (999/1218) among MSM in Guangzhou. Of the 999 HIV-positive diagnoses, 948 (95%) linked to care services, while 891 (94%) of those linked were successfully retained in care.
Ko et al. 2013	Taiwan	Cross-sectional	MSM *N* = 1037	Social media	Evaluate the effectiveness of iPOL in disseminating information about HIV, increasing the frequency of HIV testing, and reducing risky behaviours	The iPOLs actively disseminated HIV-related information via the Facebook social networking website and discussed and responded to questions from Internet-using MSM. MSM who visited the intervention or control website were surveyed after 6 months.	Frequency of online discussion or accessing information about HIV Incidence of HIV testing and condom use	MSM who visited intervention website were more likely to have HIV tests within 6 months (43.89% vs. 22.31%, *p* < 0.001); consistently use condoms during anal sex with online sex partners (34.15% vs. 26.19%, *p* = 0.004); receive HIV-related information (25.49% vs.10.47%, *p* < 0.001); discuss HIV issues with others (41.88% vs. 23.79%, *p* < 0.001); review articles about HIV (90.58% vs.79.73%, *p* < 0.001); and be asked about or discuss HIV-related questions (51.11% vs. 31.78%, *p* < 0.001) than those on the control website.
*Web-based health promotion to increase HIV testing*								
Minas et al. 2012	Australia	Cross-sectional	MSM Various sample size as multiple methods of evaluation	Website	Evaluation of a communication strategy to improve the awareness and appropriate use of nPEP	The communication strategy included: • development of nPEP information pamphlet for distribution through gay websites • nPEP resources (aimed at people at risk of acquiring HIV) and the Western Australia nPEP guidelines aimed at HCWs available on Western Australia AIDS Council website • 24 h nPEP phone line	*Immediate outcomes:* • Access and use of nPEP information • Awareness of nPEP among MSM and HCWs *Ultimate outcomes:* • nPEP treatment practice and follow up testing	Significant increase in the proportion of clients tested for HIV at 3 to 4 months after the initial visit (38.8% in 2002–2005 to 51.9% in 2008–2010, *p* = 0.023). No increase in clients tested at six to seven month after the initial visit.
Pedrana et al. 2012	Australia	Cross-sectional	MSM *N* = 295	Social marketing campaign	Evaluate the impact of a social marketing campaign in 2008–2009 aimed to increase health-seeking behaviour and STI testing and enhance HIV/STI knowledge in gay men	Impact evaluation of “Drama Downunder” health promotion campaign, by surveying online sample of gay men and analysing HIV and other STI testing data from high case load clinics before, during and after the campaign.	HIV/STI testing	Compared with the pre-campaign period, 17% increase in HIV testing rate (*p* < 0.01) were observed during the initial campaign period and 27% increase (*p* < 0.01) during the continued campaign period.
Wilkinson et al. 2016	Australia	Cohort	MSM *N = 242*	Social marketing campaign	Evaluate impact of campaign on HIV sexual health testing	Impact evaluation of social marketing campaign, by surveying online sample of MSM and analysing HIV and other STI testing data from Victorian Primary Care Network for Sentinel Surveillance HIV network before, during and after the campaign.	HIV/STI testing among MSM	Though increasing HIV/STI testing trends were observed for MSM pre and post marketing period, there was insufficient evidence to significantly attribute impact exclusively to the campaign.

**Table 3:** eHealth and HIV treatment adherence

Study	Country	Study design	Study Population Sample size (*N*=)	Technology mode	Purpose of study	Study description	Study duration	Key outcome assessed of interest in this review	Results/Authors’ conclusions
*Mobile phone calls or SMS reminders to increase adherence*									
Huang et al. 2013	China	RCT	PLHIV on ART *N* = 172	Mobile phone call	Investigate the effect of a phone call intervention to promote adherence to ART and QoL among PLHIV	I=Usual care combined with bi-weekly 3-min reminder phone calls made by trained registered nurse or other health workers C=Usual care Both groups included HIV treatment-naive and treatment-experienced HIV patients.	12 weeks	Self-reported adherence^a^ QoL	No significant improvements in adherences rates in the intervention group. Significant improvements in QoL in the intervention groups for treatment-naïve HIV patients (physical health *p* = 0.003; level of independence *p* = 0.018; environment *p* = 0.002; and spirituality/religion/personal beliefs *p* = 0.021) at 3 months.
Shet et al. 2014	India	RCT	PLHIV initiating ART *N* = 631	Mobile phone call and SMS reminders	Assess whether customised mobile phone reminders would improve adherence	I=Standard care and weekly customised, interactive, automated voice reminders, and a pictorial message sent weekly to the participants’ mobile phones C=Standard care	96 weeks	Time to virological failure ART adherence measured by pill count	No significant effect of the mobile phone intervention on either time to virological failure or ART adherence at the end of two years of therapy.
Swendeman et al. 2015	India	Cohort	PLHIV *N* = 44	IVR system using mobile phones	To design, pilot and refine IVR intervention to support ART adherence	All subjects received two IVR calls daily, timed to dosing schedules with brief messages on strategies for medical, mental health and nutrition and hygiene.	4 weeks	Self reported adherence at baseline and 1 month	Self reported missed doses decreased from 39 to 18% at one month (*p* = 0.005).
Tran et al. 2013	Vietnam	Cross sectional	PLHIV *N* = 1016	Mobile phone reminders	Assess ART adherence and its determinants among PLHIV	Multi-site cross-sectional survey: Inpatients and outpatients adult PLHIV were interviewed using structured questionnaires	N/A	Self-reported medication adherence questions Questions about medication adherence self-efficacy, reasons for missing doses and adherence aids	The main devices used for adherence supports were mobile phone alarms (62.2%). In multivariate analysis, the use of reminder strategies, such as mobile phone alarms was associated with ≥95% optimal adherence (Coefficient 0.89, 95% CI 0.02 to 1.99, *p* < 0.05).
Uzma et al. 2011	Pakistan	RCT	PLHIV initiating ART *N* = 76	Mobile phone call reminders	Assess the efficacy of interventions for improving adherence to ART regimens	I = Routine counselling and weekly phone/mobile phone call reminders C = Routine counselling	8 weeks	Self-reported adherence Pill identification test; defined as ≥95% CD4 counts Viral load	Those in the intervention condition had significantly better self-reported adherence (*p* < .001) and significantly lower viral load (*p* = .012).
*Biofeedback to improve adherence*									
Sabin et al. 2010	China	RCT	PLHIV on ART *N* = 64	EDM	Determine whether EDM feedback improved ART adherence	I=Counselling using EDM feedback C=No EDM feedback Both groups included participants assessed 6 months after initiation of treatment as either ‘low adherers’ or ‘high adherers’	12 weeks pre-interventi-on 12 weeks interventi-on	Adherence measured by EDM; defined as ≥95% Markers of disease progression	At month 12 intervention, mean adherence had risen significantly (*p* = 0.003) among intervention subjects to 96.5% but remained unchanged in controls. The mean CD4 count rose by 90 cells/μl and declined by 9 cells/μl among intervention and control subjects, respectively.
Sabin et al. 2015	China	RCT	PLHIV *N* = 119	EDM including real time wireless medication communicator	Determine whether EDM feedback improved ART adherence	Subjects with optimal and suboptimal adherence randomised to intervention or control arms. I=Individualised SMS mobile phone reminders triggered by late dose taking, and data-informed counselling. C=No reminders, standard adherence counselling	12 weeks pre-interventi-on 24 weeks interventi-on	Adherence measured by EDM; defined as ≥95% Markers of disease progression	At last intervention month, the proportion of optimal adheres was significantly higher in I group 87.3% vs. 51.8% (RR for optimal adherence in month 9, I vs. C, 1.69; CI: 1.29 to 2.21, *p* < 0.001). The mean adherence during intervention period was significantly higher in I group (I vs. C: 96.3% vs. 88.9%, *p* < 0.001). Post intervention clinical outcomes not significant.
Perera et al. 2014	New Zealand	RCT	PLHIV on ART *N* = 28	Smartphone app	Examine the efficacy of a smartphone application incorporating personalised health-related visual imagery to improve adherence to ART.	*I* = 24-h medication clock and augmented version of the smartphone app which comprised a daily real-time graphical representations of the current estimated plasma concentrations of antiretroviral drugs and simulation of protection against HIV C = standard version of the smartphone app which comprised a 24-h medication clock displaying daily ART dosing schedule and allowed participants to record when they had taken their medications each day	12 weeks	Viral load Self-reported adherence Pharmacy dispensing records	Participants in the intervention group showed a significantly higher level of self-reported adherence to ART at 3 months (*p* = 0.03) and decreased viral load (*p* = 0.023). Greater usage of the extra components of the augmented application was associated with greater perceived understanding of HIV infection and increased perceived necessity for ART.

^a^Good adherence was defined as 95% or greater

**Table 4:** eHealth and HIV testing, linkage to care and treatment adherence

Study	Country	Study design	Study Population Sample size (*N*=)	Technology mode	Purpose of study	Study description	Study duration	Key outcome assessed of interest in this review	Results/Authors’ conclusions
*Internet to increase HIV testing and treatment uptake*									
Mi et al. 2015	China	Quasi-RCT	HIV positive MSM *N* = 202	Internet	Investigate efficacy of web-based intervention in increasing HIV testing and treatment uptake among MSM	I = participants were given access to online program including an information exchanges website, a bulletin board system, an individualised online counselling with trained peer educators, and an animation game C = standard of care	24 weeks	Early initiation of ART Motivating partners to receive ART	The intervention group had significant increases in motivating partners to accept HIV testing (42.3% vs 25.5%, *p* = 0.0156) compared with the control group. There were no between-group differences on receiving ART (*p* = 0.368).

*I* intervention group, *C* control group, *PI* pre-intervention group, *MSM* men who have sex with men, *SMS* short message service, *HIV* human immunodeficiency virus, *STI* sexually transmissible infections, *VCT* Voluntary Counselling and Testing, *CI* confidence interval, *iPOL* internet popular opinion leaders, *nPEP* non-occupational post-exposure prophylaxis, *HCW* health care workers, *AIDS* acquired immunodeficiency syndrome, *RCT* randomised controlled trial, *PLHIV* people living with HIV, *ART* antiretroviral therapy, *IVR* interactive voice response, *QoL* quality of life, *SMS* short message service, *N/A* not applicable, *CD*4 cluster of differentiation 4, *EDM* electronic drug monitoring, *RR* risk ratio

### Characteristics of the included studies

The studies included in the analysis were conducted in seven Asia-Pacific countries and territories: Australia (*n* = 4), China (*n* = 6), India (*n* = 2), New Zealand (*n* = 1), Pakistan (*n* = 1), Taiwan (*n* = 1) and Vietnam (*n* = 1). Target populations included MSM (*n* = 7), PLHIV (*n* = 8), and HIV positive MSM (*n* = 1). Seven of the included studies were cross-sectional, seven were randomised controlled trials (RCT) and two were cohort studies. A total of 8869 individuals were involved in the included studies. The sample size of the RCT studies varied from 28 to 613 participants. The type of devices used to deliver eHealth interventions in engaging and retaining priority populations in the HIV treatment and care cascade were mobile phone [17, 56, 57, 59, 64], smartphone [58], and wireless monitoring device [60, 65]. The technology functions used were voice functionality of mobile phone [17, 56, 57, 59], mobile phone alarm [57], interactive voice response (IVR) [64], smartphone apps [58], SMS/text messaging [51, 53], pictorial message [59], instant messaging [53], World Wide Web access [54, 55, 62, 63, 66] and web 2.0/social media [52, 62] (details in Tables 2, 3 and 4).

The sixteen included studies were catagorised into two research foci: seven investigating eHealth applications to testing and linkage to care; and nine examining eHealth applications to HIV treatment and adherence. The methodological quality of each study included in the review was assessed and summarised in Table 5. The evaluation of the methodological quality of the HIV testing and linkage to care studies rated two studies as ‘moderate*’* [51, 52], and five studies as ‘weak*’* [53–55, 62, 63]. Quality limitations in particular related to study design, data collection methods and attrition rates.

**Table 5:** Quality assessment

STUDY	Assigned design	COMPONENT RATINGS						GLOBAL RATING
		Selection bias	Study design	Confounders	Blinding	Data collection method	Withdrawals and dropouts	
*eHealth and HIV testing and linkage to care*								
Bourne et al	Cross-sectional	Moderate	Moderate	Strong	Moderate	Weak	Weak	Moderate
Zou et al	Cross-sectional	Weak	Moderate	Weak	Moderate	Weak	Weak	Weak
Cheng et al	Cross-sectional	Weak	Weak	Weak	Moderate	Weak	Weak	Weak
Ko et al	Cross-sectional	Moderate	Moderate	Strong	Moderate	Weak	Weak	Moderate
Minas et al	Cross-sectional	Weak	Moderate	Weak	Moderate	Weak	Weak	Weak
Pedrana et al	Cross-sectional	Moderate	Moderate	Weak	Moderate	Weak	Weak	Weak
Wilkinson et al	Cohort	Weak	Weak	Weak	Moderate	Weak	Weak	Weak
*eHealth and HIV treatment adherence*								
Huang et al	RCT	Moderate	Strong	Strong	Moderate	Strong	Strong	Strong
Shet et al	RCT	Moderate	Strong	Strong	Moderate	Strong	Strong	Strong
Swendeman et al	Cohort	Weak	Moderate	Weak	Moderate	Weak	Strong	Weak
Tran et al	Cross-sectional	Moderate	Weak	Weak	Moderate	Weak	Weak	Weak
Uzma et al	RCT	Moderate	Strong	Strong	Moderate	Strong	Strong	Strong
Sabin et al. 2010	RCT	Moderate	Strong	Strong	Weak	Strong	Strong	Moderate
Sabin et al. 2015	RCT	Moderate	Strong	Strong	Moderate	Strong	Strong	Strong
Perera et al	RCT	Moderate	Strong	Strong	Moderate	Strong	Strong	Strong
*eHealth and HIV testing, linkage to care and treatment adherence*								
Mi et al	RCT	Moderate	Strong	Strong	Moderate	Weak	Strong	Moderate

Five RCT studies on eHealth applications to HIV treatment and adherence rated ‘strong’ in methodological quality [17, 56, 58, 59, 65]. Two further RCT studies were rated as ‘moderate’ due to limitations in study blinding, selection bias and data collection method [60, 66]. One cross-sectional and one cohort study were rated as ‘weak’ [57, 64]. The key limitations of these studies were related to study design, data collection methods, selection bias, and attrition rates.

### eHealth applications to increase HIV testing and linkage to care

A cross-sectional study found that SMS reminders can significantly increase HIV re-testing rates (64% vs. 30%, *p* < 0.001) among HIV-negative MSM across a nine-month period. The study found those who received SMS reminders were 4.4 more likely (95% CI 3.5 to 5.5) to re-test than the comparison group [51]. Another cross-sectional study reported that instant messaging (IM),^1^ targeting MSM through partner-seeking websites, was effective in prompting one out of every four MSM contacted through IM to present for testing [53]. However, in this study, the financial incentive offered for testing was possibly a confounding factor regarding the efficacy of the IM intervention alone.

Social media campaigns were also effective in reaching MSM and encouraging testing. A cross-sectional study used internet popular opinion leaders (iPOL) to post clips and chat about HIV prevention and testing via a MSM Facebook site. MSM who had visited the site were significantly more likely to have tested for HIV in the past six months (43.89% vs. 22.31%, χ2 = 54.8, df = 1, *p* < 0.001) compared with MSM who had visited a control site [52]. A cross sectional study from China reported increased HIV testing and linkage to care following a six year implementation of internet prevention tools to link MSM to offline HIV testing and care services [62].

The review retrieved two cross-sectional studies that found HIV promotional websites can increase HIV testing. Minas et al. [54] evaluated the impact of a promotional website as part of a multi-modal communication strategy about non-occupational post-exposure prophylaxis (nPEP) for HIV prevention. The web-based health promotion led to an improvement in the proportion of clients tested for HIV at three to four months after initial visit for nPEP (38.8% in 2002–2005 to 51.9% in 2008–2010, *p* = 0.023). While there was evidence of improvement in appropriate post nPEP testing during the campaign period, the study design precluded establishing any individual impact of the website on HIV testing and prevention, as distinct from any other element of the communication strategy. Pedrana et al. [55] assessed the impact of a social marketing campaign that employed multiple advertising channels to promote a website which provided MSM-targeted information about HIV and sexual health, including testing. Compared with the pre-campaign period, Poisson regression demonstrated significant increases (*p* < 0.01) in the average number of monthly tests among HIV-negative MSM attending three high caseload MSM clinics during the initial and continued campaign periods. However, a 2016 study [63] that further evaluated this intervention using a cohort of MSM over a longer time period, found insufficient evidence to attribute increases in HIV testing exclusively to the campaign.

### eHealth applications to improve HIV treatment adherence

This review retrieved nine studies that examined the efficacy of eHealth to improve ART adherence. One cross-sectional and three RCT studies concerned the efficacy of reminders via mobile phone calls, alarms or text messages (SMS). The cross-sectional study [57] reported that the use of automated mobile phone alarms were associated with higher optimal adherence measured by a visual analogue scale (VAS) (Coefficient 0.89, 95% CI 0.02 to 1.99, *p* < 0.01).

One RCT study [17] showed significant benefit of weekly phone call reminders together with patient involvement for cue-dosing, on improving ART adherence and virological outcomes. However two other RCT studies [56, 59] did not report significant findings; Huang et al. [56] found no significant improvement in ART adherence associated with bi-weekly three minute reminder phone calls to participants, and neither did Shet et al. [59] when examining the provision of weekly customised and interactive voice call reminders and pictorial messages. This latter study was distinct from all other retrieved studies concerning eHealth and ART adherence in the sample size (*n* = 631) and the duration of the intervention (2 years) which was significantly larger and longer than any of the other studies.

A cohort study from India investigating the efficacy of Interactive Voice Response (IVR) system timed to ART dosing schedules reported significant improvements in adherence however this study was of short duration (1 month), measuring a small sample and relying on self-report as measurement of adherence [64]. A RCT study from China examining a web-based intervention reported no significant impact on ART treatment uptake among MSM, however the intervention group showed a significant increase in motivating partners to accept HIV testing [66].

This review retrieved three RCT studies that investigated the impact of biofeedback eHealth interventions on ART adherence. Perera et al. [58] investigated a biofeedback intervention where participants received daily real-time graphical representations of estimated ART plasma concentrations via a smart phone app. They found that the intervention group had significantly higher self-reported adherence scores (48.9 vs. 47.1, *F*(1.23) = 5.37, *p* = 0.03, ηp^2^ = 0.19) and lower viral load (1.3 vs. 1.7, *F*(1.23) = 5.62, *p* = 0.023,ηp^2^ = 0.20,) at three-month follow-up when compared with the control group. Sabin et al. [60] found that the use of electronic drug monitoring (EDM) feedback and counselling led to a significant improvement in mean ART adherence at month twelve by 9.7% (86.8–96.5%, t-test statistic = − 2.84; *p* = 0.008), in the intervention group. The study also confirmed that monthly EDM-informed counselling translated into improved mean CD4 counts in the intervention compared with the control arm (rose by 90cells/μl vs. declined by 9 cells/μl, t-test statistic = − 2.4; *p* = 0.02). In a subsequent RCT study by the same research team [65], the original EDM intervention was enhanced to include real time wireless medication adherence communication. In the intervention group, optimal adherence was significantly higher at 6 months intervention (87.3% vs. 51.8%, *p* < 0.001), so too was mean adherence throughout the intervention period (96.2% vs. 89.1%, *p* = 0.003). Interestingly, clinical outcomes were not maintained post intervention.

With regard to any potential negative impact of the eHealth intervention, Shet et al. [59] noted that although participants did not report adverse events during the study, six of 286 participants interviewed, expressed concerns about intrusiveness and loss of privacy associated with the intervention.

## Discussion

To our knowledge, this is the first systematic review of the evidence for the application of eHealth to improve linkage to and retention in HIV clinical care in the Asia-Pacific region. We acknowledge that similar approaches have been applied to other disease areas such as cardiovascular disease and diabetes [67, 68]. However, for HIV this systematic review is significant in that it synthesises all the important findings related to eHealth for HIV, across the HIV treatment and care cascade. This review found weak to moderate evidence to support the use of eHealth, via text messages, instant messaging, social media and health promotion websites, to increase rates of HIV testing and re-testing among MSM. Evidence regarding the efficacy of eHealth interventions to improve ART adherence using text messages or mobile phone call reminders was mixed. Two strong quality studies [56, 59] found no significant effect, whereas one strong quality study [17] and two weak quality studies [57, 64] reported significant findings. Innovative moderate to strong quality studies where participants received individualised biofeedback about their estimated ART plasma concentration levels, have shown promising results for ART adherence [58, 60, 65].

**Fig. 2:**
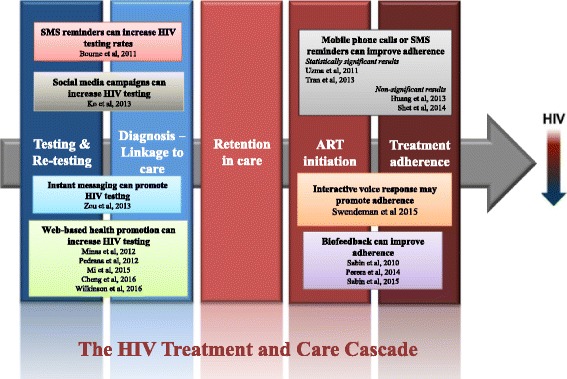
mHealth interventions across the HIV treatment and care cascade

As can be seen in Fig. 2, this review revealed that a number of studies have shown the usefulness of eHealth tools to facilitate testing, re-testing and ART adherence, however there were no studies investigating the efficacy of eHealth in retention in care. There is much potential to incorporate eHealth interventions to fill the gaps of the HIV treatment and care cascade to reduce rates of loss to follow-up, thus improve retention in HIV care [69]. Retention in care, starting from a positive HIV test to enrolment in care, initiation of ART and continuation of lifelong ART, is critical to ensure the best possible outcomes for PLHIV, reduce development of ART resistance and reduce the incidence of new infections [70].

The findings of the current review have several important implications for future interventions. In October 2014 the Joint United Nations Programme on HIV/AIDS (UNAIDS) published *90–90-90*, an HIV treatment strategy to end the AIDS epidemic. Specifically, the targets of this strategy are that by 2020: 90% of all PLHIV will know their HIV status; 90% of all people diagnosed with HIV will receive sustained ART; and 90% of all people receiving ART will have viral suppression [71]. In a world where the development and expansion of digital technology are growing exponentially, eHealth interventions can have an important role to play in the movement towards the end of AIDS. Regarding the first target of the UNAIDS 90–90-90 strategy, the findings of this review lend support to the effectiveness of SMS reminders, IM and social media campaigns, at increasing HIV testing among MSM, particularly young MSM who are typically early adopters of new technologies [72]. These types of intervention warrant further attention and application in particular among other priority populations who may be marginalised and thus harder-to-reach, such as SW, PWID and TG, especially given that eHealth interventions are evidenced to be feasible and acceptable to these groups [21–33]. Concerning the second and third strategy targets, treatment and viral suppression, there is mixed evidence for the efficacy of SMS and mobile phone call reminders to improve adherence. Promising results regarding the efficacy of individualised real-time biofeedback merit further investigation to determine whether such intervention could be further enhanced and cost-effectively rolled out. Furthermore, there remains a lack of longer term and larger scale clinical outcome studies to investigate whether behavioural changes are maintained over time and post intervention.

In line with the recent 2016 *WHO Consolidated guidelines on the use of antiretroviral drugs for treating and preventing HIV infection: Recommendations for a public health approach* [73] and the 2015 UNFPA *Implementing comprehensive HIV and STI programmes with men who have sex with men* [35] publications, we recommend that future HIV strategies, policies and planning documents should include guidelines for incorporating eHealth in prevention, testing and treatment programmes.

eHealth can also contribute to the efficacy of pre-exposure prophylaxsis (PrEP) intervention programmes. There is growing evidence for the role of eHealth interventions in assisting in the rollout of PrEP in priority populations [74]. This information is pertinent to the Asia-Pacific region where countries would benefit from including PrEP in their HIV prevention programmes. HIV testing and re-testing will be very important for increasing uptake of PrEP and also PrEP adherence and prospective evaluations of these interventions should include countries in Asia-Pacific region [75].

In utilising eHealth technologies in HIV programmes and services, it is important that steps are taken to ensure the digital security of any client information collected. Confidentiality and privacy concerns are justifiably heightened among PLHIV given the stigma attached to this diagnosis especially in low and middle income countries where mobile phones may be shared among families and communities. We recommend that future eHealth interventions in HIV take steps to ensure the digital security of confidential information so that PLHIV and people at risk of HIV can confidently engage with these interventions. The WHO GOe list the following parameters that should be included in eHealth security protocols: password protection; network log in requirements; synchronisation of files to the device; and data backup and recovery [5]. The UNFPA 2015 guideline *Implementing comprehensive HIV and STI programmes with men who have sex with men* recommends consulting technical experts in ICT security where possible to develop effective security protocols [35]. In line with the Greater Involvement of People Living with HIV (GIPA) principle, consultation with PLHIV in the development of eHealth security protocols should also be prioritised [76].

Although there is emerging evidence to support the use of eHealth in improving HIV outcomes, it is important to consider potential risks associated with the intrusiveness of mobile technologies. In a Kenyan study on SMS reminders to promote ART adherence, Pop-Eleches et al. [77] found that daily text message reminders did not improve ART adherence more than weekly reminders and posited that daily messages were considered intrusive by participants. Similarly, in a meta-analysis of eight RCTs to promote HIV medication adherence, Finitsis et al. [12] noted that increased frequency of daily text messaging showed smaller effects in participants’ adherence than interventions that messaged multiple times a week or weekly. Perceptions of intrusiveness will need to be examined further in future eHealth studies, to reduce possible negative impacts of mobile technologies, and maximise optimal outcomes for eHealth interventions.

The findings of this systematic review are inevitably limited by the scope and quality of the original quantitative research. Employing qualitative studies in a systematic review will provide a more in-depth perspectives of the target group of an intervention [78], whether such intervention is feasible, meaningful and appropriate to the target group [79]. An important aspect of evidence synthesis to inform policy and practice is including diverse forms of evidence from different types of research to maximise the findings [80]. No qualitative studies were included in this review, however a mixed method approach should be considered in future systematic reviews.

This systematic review was broad in its scope enabling the inclusion of a variety of technology interventions to address the HIV testing, care, and treatment among HIV priority populations. Other strengths of this review include a comprehensive and sensitive search strategy that covered all types of eHealth across numerous electronic databases aiming to identify all published and unpublished literature. However, this systematic review has certain limitations. First, although six of the retrieved studies were conducted in China, the restriction of this systematic review to English language articles may have excluded non-English language literature from other Asia-Pacific countries which may have omitted some relevant articles. All of the selected studies were from Asian countries, Australia or New Zealand, with none from Pacific Island countries or territories. Due to the variability of the study characteristics, study designs, type of eHealth interventions and outcome measures, it was not feasible to perform a meta-analysis. All eight of the studies concerning eHealth applications to HIV testing and linkage to care, were rated either weak or moderate in methodological quality, limiting the strength of any findings reported. And finally, all but one of the retrieved studies that investigated treatment and adherence described participants primarily as PLHIV, without further delineation according to priority population groups. This makes drawing conclusions about the efficacy of eHealth in HIV treatment and adherence specifically for priority populations, limited.

## Conclusions

In summary, our review demonstrates encouraging evidence about how eHealth can be used across the HIV treatment and care cascade, including increasing HIV testing in priority populations and ART adherence in the Asia-Pacific region. Moreover the application of eHealth to increasing the uptake of PrEP in priority populations, should also be investigated. These findings can inform HIV programme planning and service delivery regarding the incorporation of eHealth to enhance HIV prevention, testing, treatment, and care. Such interventions are relatively cost-effective, and acceptable to harder to engage priority populations at higher risk of HIV and of dropping out of the HIV treatment and care cascade. The use of eHealth is particularly applicable in the Asia-Pacific region where the internet and mobile phone uptake is high.

## Additional file

Additional file 1: Demonstration search strategy for PubMed. (DOC 35 kb)

## Abbreviations

AIDS: acquired immunodeficiency syndrome
Apps: mobile phone applications
ART: antiretroviral treatment
BHIVA: British HIV/AIDS Association
CD4: cluster of differentiation 4
CINAHL: Cumulative Index to Nursing and Allied Health Literature
EDM: electronic drug monitoring
GIPA: Greater Involvement of People Living with HIV
GOe: Global Observatory for eHealth
HIV: Human immunodeficiency virus
ICT: information and communication technologies
IM: instant messaging
iPOL: internet popular opinion leaders
IVR: interactive voice response
MeSH: medical subject heading
MSM: men who have sex with man
nPEP: non-occupational post-exposure prophylaxis
PDA: personal digital assistant
PICO: Population, Intervention, Comparison and Outcomes
PLHIV: people living with HIV
PrEP: pre-exposure prophylaxis
PRISMA: Preferred Reporting Items for Systematic Reviews
PWID: people who inject drugs
RCT: randomised controlled trial
SMS: short message service
STI: sexually transmissible infections
SW: sex workers
TG: transgender
UNAIDS: Joint United Nations Programme on HIV/AIDS
UNFPA: United Nations Population Fund
VAS: visual analogue scale
WHO: World Health Organization
WHOLIS: World Health Organization Library Information System

## Footnotes

1 The exchange of text messages through a software application in real-time
