# Supplementary material for: Using eHealth to engage and retain priority populations in the HIV treatment and care cascade in the Asia-Pacific region: a systematic review of literature
Source: BMC Infect Dis. 2018 Feb 17;18:82. doi: 10.1186/s12879-018-2972-5 (PMC5816561; doi:10.1186/s12879-018-2972-5)
Supplement: Supplementary file 1 — Demonstration search strategy for PubMed. (DOC 35 kb) [file 12879_2018_2972_MOESM1_ESM.doc]

**Additional file 1: Demonstration search strategy for PubMed**

**HIV testing and linkage to care**

| **Search** | **Query** |
| --- | --- |
| #1 | HIV[MH] OR HIV Infection[MH] OR hiv[T/A] OR hiv-1*[T/A] OR hiv-2*[T/A] OR hiv Infect*[T/At] OR (human immunodeficiency virus OR human immunedeficiency virus)[T/A]) OR acquired immunodeficiency syndrome[MH] OR acquired immunedeficiency syndrome[T/A] |
| #2 | Computer[MH] OR handheld computer[MH] OR tablet PC[T/A]) OR Playstation Portable[T/A] OR playstation[T/A] OR Mobile phone[T/A] OR Cellular Phone[MH] OR Cell Phone[T/A] OR hand phone[T/A] OR Palm Pilot[T/A] OR PDA phone[T/A] OR BlackBerry[T/A] OR Personal digital assistant[T/A] OR Pedometer[T/A] OR Personal computer[T/A] OR Desktop[T/A] OR laptop[T/A) OR PC[T/A] OR Pocket PC[T/A] OR MP3-Player[MH] OR MP4-Player[T/A] OR Smartphone[T/A] OR Hub[T/A]OR Blogging[MH] OR podcasts[T/A] OR Email[T/A] OR e-mail[T/A] OR electronic mail[MH] OR instant messaging[T/A) OR Internet[MH] OR Chat Room[T/A] OR live chat[T/A] OR mHealth[T/A] OR MMS[T/A]OR SMS[T/A] OR Text Messaging[MH] OR Multimedia[MH] OR Netbook*[T/A) OR Online[T/A] OR Reminder Systems[MH] OR Social Media[MH] OR Facebook[T/A] OR Twitter[T/A] OR Myspace[T/A] OR YouTube[T/A]) OR Social Networking[MH]) OR Internet forums[T/A] OR teleHealth[T/A] OR Telemedicine[MH] OR eHealth[T/A] OR Mobile Health[T/A] OR Web based[T/A] OR Wireless Technology[MH] OR Wi-Fi[T/A] OR World wide web[T/A] OR world-wide-web[T/A] OR website[T/A] OR web-site[T/A] OR Mobile Applications[MH] |
| #3 | Test*[T/A] OR screen*[T/A] OR counsel*[T/A] OR diagnose*[T/A] |
| #4 | commercial sex work*[T/A] OR csw*[T/A] OR *male sex worker*[T/A] OR fsw*[T/A] OR ( ( money OR paid ) W/2 sex )[T/A] OR prostitut*[T/A] OR ( sex n/4 money )[T/A] OR sexwork*[T/A] OR sex-work*[T/A] OR transactional sex[T/A] OR gay*[T/A] OR ( gay PRE/0 ( m?n OR male ) )[T/A] OR ( homosexual* PRE/0 ( m?n OR male ) ) [T/A] OR homosexuality, male[MH] OR men who have sex with men[T/A] OR msm*[T/A] OR ( sex with PRE/0 ( m?n OR male ) )[T/A] OR bisexual men[T/A] OR bisexuality[MH] OR ( men who have sex with men AND women )[T/A] OR male to male sex[T/A] OR queer [T/A] OR intravenous drug abuse[T/A] OR ( ( drug OR substance* ) W/2 ( abuse* OR *use* OR dependent* OR disorder* OR addict* ) )[T/A] OR idu*[T/A] OR ( ( illicit OR recreational ) W/1 drug users )[T/A] OR injecting drug use*[T/A] OR injecting substance[T/A] OR ivdu[T/A] OR needle sharing[T/A] OR people who inject drugs [T/A] OR pwid[T/A] OR substance abuse, intravenous[MH] OR ( intravenous PRE/0 ( drug OR substance ) ) [T/A] OR bigender[T/A] OR drag queen[T/A] OR female-to-male[T/A] OR gender ambiguous[T/A] OR intersex*[T/A] OR male-to-female[T/A] OR transgender*[T/A] OR transgendered persons[MH] OR transsexualism[MH] OR transman[T/A] OR transsexual[T/A] OR trans-sexual[T/A] OR transsexual person*[T/A] OR transvestite[T/A] OR transwoman[T/A] |
| #5 | #1 AND #2 AND #3 AND #4 |

**HIV treatment, adherence and retention in care**

| **Search** | **Query** |
| --- | --- |
| #1 | HIV[MH] OR HIV Infection[MH] OR hiv[T/A] OR hiv-1*[T/A] OR hiv-2*[T/A] OR hiv Infect*[T/At] OR (human immunodeficiency virus OR human immunedeficiency virus)[T/A]) OR acquired immunodeficiency syndrome[MH] OR acquired immunedeficiency syndrome[T/A] |
| #2 | Computer[MH] OR handheld computer[MH] OR tablet PC[T/A]) OR Playstation Portable[T/A] OR playstation[T/A] OR Mobile phone[T/A] OR Cellular Phone[MH] OR Cell Phone[T/A] OR hand phone[T/A] OR Palm Pilot[T/A] OR PDA phone[T/A] OR BlackBerry[T/A] OR Personal digital assistant[T/A] OR Pedometer[T/A] OR Personal computer[T/A] OR Desktop[T/A] OR laptop[T/A) OR PC[T/A] OR Pocket PC[T/A] OR MP3-Player[MH] OR MP4-Player[T/A] OR Smartphone[T/A] OR Hub[T/A]OR Blogging[MH] OR podcasts[T/A] OR Email[T/A] OR e-mail[T/A] OR electronic mail[MH] OR instant messaging[T/A) OR Internet[MH] OR Chat Room[T/A] OR live chat[T/A] OR mHealth[T/A] OR MMS[T/A]OR SMS[T/A] OR Text Messaging[MH] OR Multimedia[MH] OR Netbook*[T/A) OR Online[T/A] OR Reminder Systems[MH] OR Social Media[MH] OR Facebook[T/A] OR Twitter[T/A] OR Myspace[T/A] OR YouTube[T/A]) OR Social Networking[MH]) OR Internet forums[T/A] OR teleHealth[T/A] OR Telemedicine[MH] OR eHealth[T/A] OR Mobile Health[T/A] OR Web based[T/A] OR Wireless Technology[MH] OR Wi-Fi[T/A] OR World wide web[T/A] OR world-wide-web[T/A] OR website[T/A] OR web-site[T/A] OR Mobile Applications[MH] |
| #3 | ART[T/A] OR Antiretroviral[T/A] OR Therapy[T/A] OR Medication[T/A] OR resist*[T/A] OR PEP*[Title/A] OR Prophylaxis[T/A] OR treatment[T/A] OR PrEP[T/A] OR retent*[T/A] OR retain*[T/A] OR "in care"[T/A] OR link*[T/A] OR "follow up"[T/A] OR adherence[T/A] OR cascade[T/A] OR suppress*[T/A] |
| #4 | #1 AND #2 AND #3 |
